# Antioxidant Capacity and Radical Scavenging Effect of Polyphenol Rich *Mallotus philippenensis* Fruit Extract on Human Erythrocytes: An *In Vitro* Study

**DOI:** 10.1155/2014/279451

**Published:** 2014-11-26

**Authors:** Mayank Gangwar, Manish Kumar Gautam, Amit Kumar Sharma, Yamini B. Tripathi, R. K. Goel, Gopal Nath

**Affiliations:** ^1^Department of Pharmacology, Faculty of Modern Medicine, Institute of Medical Sciences, Banaras Hindu University, Varanasi 221005, India; ^2^Department of Microbiology, Faculty of Modern Medicine, Institute of Medical Sciences, Banaras Hindu University, Varanasi 221005, India; ^3^Department of Medicinal Chemistry, Institute of Medical Sciences, Banaras Hindu University, Varanasi 21005, India

## Abstract

*Mallotus philippinensis* is an important source of molecules with strong antioxidant activity widely used medicinal plant. Previous studies have highlighted their anticestodal, antibacterial, wound healing activities, and so forth. So, present investigation was designed to evaluate the total antioxidant activity and radical scavenging effect of 50% ethanol fruit glandular hair extract (MPE) and its role on Human Erythrocytes. MPE was tested for phytochemical test followed by its HPLC analysis. Standard antioxidant assays like DPPH, ABTS, hydroxyl, superoxide radical, nitric oxide, and lipid peroxidation assay were determined along with total phenolic and flavonoids content. Results showed that MPE contains the presence of various phytochemicals, with high total phenolic and flavonoid content. HPLC analysis showed the presence of rottlerin, a polyphenolic compound in a very rich quantity. MPE exhibits significant strong scavenging activity on DPPH and ABTS assay. Reducing power showed dose dependent increase in concentration absorption compared to standard, Quercetin. Superoxide, hydroxyl radical, lipid peroxidation, nitric oxide assay showed a comparable scavenging activity compared to its standard. Our finding further provides evidence that *Mallotus* fruit extract is a potential natural source of antioxidants which have a protective role on human Erythrocytes exhibiting minimum hemolytic activity and this justified its uses in folklore medicines.

## 1. Introduction

Free radical and reactive oxygen species (ROS) are basically the main cause of several disorders in humans that are generated as an imbalance between formation and neutralization of prooxidants resulting in oxidative stress. They cause oxidative damage to lipids, proteins, and DNA, eventually leading to many chronic diseases, such as cancer, diabetes, aging, and other degenerative diseases in humans [1, 2] along with lipid peroxidation. To protect the adverse effects of free radical, human cells generate enzymes such as superoxide dismutase (SOD) and catalase or compounds such as ascorbic acid, tocopherol, and glutathione [3].

Plants are rich in antioxidants; so much attention has been directed towards the development of ethnomedicines as they contain phenols, flavonoids, alkaloids, tannins, vitamins, terpenoids, and many more phytochemicals responsible for different pharmacological activities [4]. Current research has proofed that ingestion of natural antioxidants has been associated with reduced risk of cancer and many chronic diseases [5].

*Mallotus philippenensis *Lam. Muell. Arg (Euphorbiaceae) (MP) are shrubs or small trees which grow on mountain slopes or valleys, limestone hills or river valleys, and forests at an altitude of 300–1600 m.* Mallotus philippenensis* is well known to contain different natural compounds, mainly phenols, diterpenoids, steroids, flavonoids, cardenolides, triterpenoids, coumarins, isocoumarins and many more which exhibit interesting biological activities such as antimicrobial [6], anticestodal [7, 8], antiviral [9], immune-regulatory, antiviral, or cytotoxic along with being also used as purgative, anthelmintic, vulnerary, detergent, maturant, carminative, alexiteric and being useful in treatment of bronchitis, abdominal diseases [10].

The objective of the present investigation was to evaluate the total antioxidant potential and radical scavenging activity of a 50% ethanol extract of* Mallotus philippenensis* fruit. The extract was examined for different ROS scavenging activities including hydroxyl, superoxide, hydroxyl radical, nitric oxide, and lipid per oxidation. Phenol and flavonoid contents were determined which may be responsible for antihemolytic effect tested* in vitro* on human erythrocytes. Oxidative damage by ROS and free radicals can be protected by phenolic compounds, as they act as exogenous antioxidant system mainly from diet [11]. ROS are involved in the mechanism that can contribute to metabolic disorders and endothelial dysfunction [12]; therefore, natural antioxidants compounds rich in phenol and flavonoids could reverse these damages. The exact molecular mechanisms of action of phenols, flavonoids, and other antioxidants have not yet fully been elucidated and are still a matter of considerable research. Total antioxidant activity of plant extract was evaluated by standard methods of DPPH and ABTS assay.

## 2. Material and Methods

### 2.1. Chemicals and Instruments

Ferric chloride, potassium ferricyanide, potassium persulfate, trifluoroacetic acid (TFA), and trichloroacetic acid (TCA) were acquired from the P.O.Ch. Company (Gliwice, Poland). (+)-Catechin, (−)-epicatechin, Folin and Ciocalteau's phenol reagent, Dragendroff's reagent, Hager's reagent, Mayer's reagent, Wagner's reagent, Griess reagent, Fenton reagent, sodium picrate, ninhydrin solution, quercetin, gallic acid, 2,2-diphenyl-1-picrylhydrazyl radical (DPPH), and 2,2′-azinobis-(3-ethylbenzothiazoline-6-sulfonic acid) (ABTS) were purchased from Sigma Ltd. (Poznań, Poland). Ascorbic acid, nitroblue tetrazolium (NBT), ethylenediamine tetraacetic acid (EDTA), nitro blue tetrazolium (NBT), reduced nicotinamide adenine dinucleotide (NADH), phenazine methosulfate (PMS), thiobarbituric acid (TBA), thiourea, thiobarbituric acid reactive substances (TBARS), aluminium chloride (AlCl_3_), sodium nitroprusside (SNP), sulfanilamide, naphthylethylenediamine dihydrochloride (NED), and butylated hydroxytoluene (BHT) were obtained from HiMedia Laboratories Pvt., Ltd, Mumbai, India.

### 2.2. Plant Material and Preparation of Extract

*Mallotus philippenensis* fruits were collected from Botanical Garden, Department of Dravyaguna, Institute of Medical Sciences, Banaras Hindu University (25.5°N, 82.9°E; elevation, 79 ft/85 m), India. The plant was collected in March to April during fruiting season and was identified and authenticated by Professor R. K. Asthana Department of Botany, Banaras Hindu University, India. A reference voucher number RKA/BOT/Sept. 10–12 was assigned to the plant samples and preserved in Department of Botany. The red color glandular hair powder adhering at the surface of shade dried fruits was collected. Approximately 500 g of powder was added to 1000 mL of 50% ethanol in a round bottom flask and was kept at room temperature for 3 days in shade. The organic fraction was collected and concentrated invacuum in a rotary evaporator and the residue was dried in desiccators over calcium chloride.

### 2.3. Qualitative Phytochemical Estimation

The crude 50% ethanolic extract of glandular hairs of* Mallotus* fruit (MPE) was examined by standard methods and preliminary study was carried out by chemical test.

#### 2.3.1. Tests for Carbohydrates

*(a) Molisch's Test*. Solutions of extracts were mixed with few drops of Molisch's reagent (*α*-Naphthol) and conc. sulfuric acid was added from side of test tube. Formation of purple color ring at junction indicated the presence of carbohydrates [13].

*(b) Fehling Solution Test*. One mL each of Fehling A and Fehling B solutions were mixed with 2 mL of different extracts. The mixtures were boiled for 5–10 minutes on water bath. Reddish brown color was obtained due to formation of cuprous oxide which indicated the presence of reducing sugar [13].

#### 2.3.2. Tests for Alkaloids

*(a) Dragendroff's Test*. One mL of Dragendroff's reagent was added to different extracts. Formation of reddish brown precipitate indicated the presence of alkaloids.

*(b) Mayer's Test*. One mL of Mayer's reagent was added to different extracts. Formation of cream color precipitate indicated the presence of alkaloids.

*(c) Wagner's Test*. One mL of Wagner's reagent was added to different extracts. Formation of reddish brown precipitate indicated the presence of alkaloids.

*(d) Hager's Test*. One mL of Hager's reagent was added to different extracts. Formation of yellow colour precipitate indicated the presence of alkaloids.

#### 2.3.3. Tests for Fats and Fixed Oils

Five drops of different extract samples were treated with 1% copper sulphate solution and then 10% sodium hydroxide solution was added. Appearance of clear blue solution indicated the presence of fats and fixed oils.

#### 2.3.4. Tests for Flavonoids

*(a) Alkaline Reagent Test*. To the extract samples few drops of sodium hydroxide solution were added. Formation of intense yellow color, which turned colorless after addition of few drops of dilute hydrochloric acid, indicated the presence of flavonoids.

*(b) Shinoda Test*. To the extract samples few magnesium turnings and few drop of conc. hydrochloric acid were added after few minutes' appearance of crimson red color indicated the presence of flavonoids.

#### 2.3.5. Tests for Anthraquinone Glycosides

*Borntrager's Test*. Different extract samples were boiled with 1 mL of sulfuric acid in a test tube for 5 minutes and filtered while hot. Filtrate was cooled and shaken with equal volume of chloroform. Lower layer of chloroform was separated and it was shaken with half of its volume of dilute ammonia. Formation of rose pink to red colour in the ammonical layer indicated the presence of anthraquinone glycosides [13].

#### 2.3.6. Tests for Cardiac Glycosides

*(a) Keller-Kiliani Test (Test for Deoxy Sugars)*. This test was carried out by extracting the drug with chloroform and the extract was evaporated to dryness then 0.4 mL glacial acetic acid containing trace amount of ferric chloride was added. After transferring to a small test tube, 0.5 mL of conc. sulfuric acid was added by the side of test tube. Appearance of blue color of acetic acid layer indicated the presence of cardiac glycosides.

*(b) Legal Test*. The extract samples were treated with pyridine and then alkaline sodium nitroprusside solution was added. Appearance of blood red color indicated the presence of cardiac glycosides.

*(c) Baljet Test*. The extract samples were treated with sodium picrate. Appearance of orange color indicated the presence of cardiac glycosides.

#### 2.3.7. Tests for Saponin Glycosides

*Froth Formation Test*. Two mL of each extract sample was placed with water in a test tube and shaken well. Formation of stable froth (foam) indicated the presence of saponin glycosides.

#### 2.3.8. Tests for Tannins

*(a) Ferric Chloride Test*. Different extract samples were treated with ferric chloride solution; appearance of blue and green colors indicated the presence of hydrolysable and condensed tannins, respectively [14].

*(b) Gelatin Test*. 1% gelatin solution containing 10% sodium chloride was added to different extract samples. Formation of precipitate indicated the presence of tannins [14].

#### 2.3.9. Tests for Proteins

*(a) Heat Test*. Different extract samples were heated on a boiling water bath; coagulation of samples indicated the presence of proteins.

*(b) Hydrolysis Test*. Different extract samples were hydrolyzed with hydrochloric acid and then ninhydrin solution was added and boiled. Appearance of violet colour indicated the presence of proteins.

#### 2.3.10. Tests for Steroids and Triterpenoids

*Salkowski's Test*. Different extract samples were treated with few drops of concentrated sulfuric acid. Appearance of red and yellow color at the lower layer indicated the presence of steroids and triterpenoids, respectively [15, 16].

### 2.4. TLC and HPLC Profiling of MPE

#### 2.4.1. Thin Layer Chromatography (TLC) Fingerprinting

Thin layer chromatography (TLC) was used to separate the different part of MPE into different spots on the chromatoplate spotted on silica gel precoated TLC plate and allowed to rise in different solvent systems in saturated TLC chamber. The chromatograms developed on the microscope slide were dried and observed visually for the various different spots of plant extract. The developing solvent used in extract is chloroform and methanol with ratio 9 : 1. Their *R*_*f*_ values were recorded as the ratio of distance traveled by spots to the distance traveled by solvent system as described by Kajaria et al., 2011. The retention factor was calculated using

(1) $$\begin{array}{c} R_{f}=\frac{\text{Distance}\;\;\text{move}\;\;\text{by}\;\;\text{the}\;\;\text{substance}\;\left(\text{cm}\right)}{\text{Distance}\;\;\text{move}\;\;\text{by}\;\;\text{the}\;\;\text{solvent}\;\left(\text{cm}\right)}. \end{array}$$

#### 2.4.2. High Performance Liquid Chromatography (HPLC)

For HPLC analysis, 50 mg crude extract was weighed and diluted to volume with methanol in a 2.0 mL volumetric flask. Then, the solution was mixed during 15 min on a shaking bath (Narang Scientific, New Delhi) and filtered through a filter of Schleicher and Schuell (Dassel, Germany) with a diameter of 125 mm and a pore size smaller than 2 *µ*m. A sample of alcoholic fruit extract (MPE) was analyzed without any treatment.

Separation for qualitative and quantitative analysis of flavonoids was performed by HPLC-PDA with a LC-10 (Shimadzu, Japan) system comprising an LC-10AT dual pump, an SPD-M20A PDA detector, and rheodyne injection valve furnished with a sample loop (20 mL). Molecules were separated on a 250 mm × 4.6 (i.d.), 5 mm pore size RP-C18 column (Merck) protected by guard column containing the same packing. The mobile phase was a gradient prepared from 0.5% (v/v) phosphoric acid in HPLC-grade water (component A) and methanol (component B). Before use, the components were filtered through 0.45 mm nylon filters and deaerated in an ultrasonic bath. The gradients from 25 to 50% B in 0–3 min, 50 to 80% B in 3–18 min, 80 to 25% B in 25 min, and 25% B in 30 min were used for conditioning of the column with a flow rate of 0.8 mL/min. Data were integrated by Shimadzu class VP series software and quantification was carried out by comparison with standards. Quantitative values are mean values from three replicate analyses of the same sample extracted in three different points. All samples and solutions were filtered through 0.45 mm nylon filters (Millipore, USA) before analysis by HPLC. Simple mobile phase was used as control for identification of blank peaks. The chromatogram was compared with the chromatogram of standards.

### 2.5. Quantitative Estimation of MPE

#### 2.5.1. Estimation of Total Phenolic Content

The total phenolic content in plant material was determined according to the Singleton and Rossi Jr. method, with some modifications [17]. Estimation was done by folin-ciocalteu method, where phenolic compounds form a blue complex. The gallic acid was used as standard solution. 0.5 mL of test extract solution was mixed with 2.5 mL of 1 N folin-ciocalteu reagent and incubated for 5 minutes and then 2 mL of 75 g/L sodium carbonate was added followed by distilled water. After incubation at room temperature for 2 hours, absorbance of reaction mixture was measured at 760 nm against blank as methanol. The total phenolic content was expressed in *µ*g of gallic acid equivalent (GAE) of dry plant material. The linearity range for this assay was determined as 0.5–5.0 mg/L GAE (*R* ^2^ = 0.999), giving an absorbance range of 0.050–0.555 absorbance units [18–20].

#### 2.5.2. Estimation of Total Flavonoid Content

Total flavonoid content in plant material was determined colorimetrically according to the method described by Lamaison and Carret [21] by using quercetin as standard. Here 5 mL of 2% aluminum chloride in methanol was mixed with the same volume of test solution, after incubation of 10 minutes; absorbance was measured at 415 nm against blank sample. The total flavonoid content was determined using a standard curve of quercetin at 0–50 *µ*g/mL. The average of three readings was used and then expressed in *µ*g quercetin equivalent flavones per mg extract [20].

#### 2.5.3. Estimation of Antioxidant Activity

*(a) DPPH Radical Scavenging Activity*. DPPH inhibition in MPE was determined by using the protocol of Brand-Williams et al., [22] with some modifications [23]. The DPPH radical (Hi-media) is stable due to the delocalization of a spare electron over the molecule, thus preventing dimer formation. This radical is used in the DPPH radical scavenging capacity assay to quantify the ability of antioxidants to quench the DPPH radical. The dark purple color of DPPH will be lost when it is reduced to its nonradical form stable organic nitrogen centered free radical with a dark purple color which when reduced to its nonradical form by antioxidants becomes colorless. DPPH radicals are widely used in the model system to investigate the scavenging activities of several natural compounds. When the DPPH radical is scavenged, the color of the reaction mixture changes from purple to yellow with decreasing of absorbance at wavelength 517 nm. 200 mg of sample was taken in centrifuge tube (in triplicate). Two hundred microliter distilled water was taken in blank instead of the sample. Then 1 mL of DPPH (8 mg/100 mL of ethanol) solution was added to the sample and the blank. This setup was left at room temperature for 30 minutes (vertexed in between). Tubes were then centrifuged at 4000 rpm for 10 min. After that, 0.5 mL supernatant was poured in fresh tubes containing 1 mL of ethanol (ethanol absolute 99.9%, analytical reagent, Changshu Yangyuan Chemical, China) and the absorbance was taken at 517 nm against the ethanol by using UV-1800 spectrophotometer (Shimadzu, Japan). Each crude extract was analyzed in triplicate. The percentage of inhibition was calculated against blank:

(2) $$\begin{array}{c} I\%=\frac{A\;\;\text{blank}-A\;\;\text{sample}}{A\;\;\text{blank}}\times 100, \end{array}$$

where *A*_blank_ is the absorbance of the control reaction (containing all reagents except the test compound) and *A*_sample_ is the absorbance of the test compound.

*(b) Total Antioxidant/ABTS Radical Scavenging Activity*. ABTS^∗+^ radical-scavenging activity of MPE was determined according to Re et al. [24] ABTS^∗+^ radicals were pregenerated by adding 5 mL of a 4.9 mM potassium persulfate solution to 5 mL of a 14 mM ABTS solution and kept for 16 h in the dark. Different concentrations of extract (50–700 *μ*g/mL) were added to the above activated pregenerated ABTS solution. This solution was suitably diluted with distilled water to yield an absorbance of 0.70 at 734 nm and then used for antioxidant assay. Ascorbic acid (50 *µ*g/mL) was used as reference compound. 50 *μ*L was added to 950 *μ*L of ABTS solution and vortexed for 10 s and after 6 min and then reduction in absorbance was recorded at 734 nm, using distilled water as a blank, on ELICO (SL-150) UV-visible spectrophotometer (Sanathnagar, Hyderabad, Andhra Pradesh, India). Same volume of test solutions of each extract was also taken in similar manner. The result was compared with control (only ABTS solution) having absorbance 0.712 ± 0.032 [24].

*(c) Superoxide Radical Scavenging Activity*. Super oxide radical scavenging potential of different fractions of MPE was reported in terms of its capacity to inhibit the formazan formation upon photochemical reduction of nitroblue tetrazolium (NBT) [25]. In brief, each 3 mL reaction mixture (0.01 M phosphate buffer (pH 7.8), 130 mM methionine, 60 *μ*M riboflavin, 0.5 mM EDTA, NBT (0.75 mM) with 0.5 mL extract/CuSO_4_ solution; positive Control). These tubes were kept in front of fluorescent light for 6 minutes and absorbance was taken at 560 nm. The nonenzymatic phenazine methosulfate-nicotinamide adenine dinucleotide (PMS-NADH) system generates superoxide radicals, which reduce NBT to a purple formazan. The decrease in absorbance at 560 nm with the plant extract and the reference compound quercetin indicates their abilities to quench superoxide radicals in the reaction mixture. Identical tubes were kept in the dark and served as blanks. The results were expressed in percent inhibition as compared to control.

*(d) Hydroxyl Radical Scavenging Activity*. The deoxyribose method described by Aruoma and Halliwell [26] was used to determine the hydroxyl radicals trapping capacity of MPE, as per standard method. Here FeCl_3_-EDTA-ascorbic acid was used to generate OH radicals, as detailed below. The reaction was carried out in 2 conditions, that is, in presence of EDTA, (non-site-specific) to determine its OH trapping capacity and in absence of EDTA (site specific) to assess its metal chelation property.

This experiment was performed to check the effect of MPE on hydroxyl (OH.) radical's trapping potential. Different concentrations (50–700 *μ*g/mL) of extract were added to the reaction mixture in a final volume of 1 mL in potassium phosphate buffer (10 mM, pH 7.4). This mixture was incubated at 37°C for 1 h and then mixed with 1 mL of 2.8% TCA (w/v in water) and 1 mL of 1% thiobarbituric acid (TBA) (w/v). It was then heated in a boiling water bath for 15 min and cooled and absorbance was taken at 532 nm. Here, thiourea was taken as positive control. Thiourea was used as standard as hydroxyl radical scavenger. The above experiment was repeated in absence of EDTA to assess the metal chelation property of plant extract. The difference between 2 readings (absorbance in presence and absence of EDTA) at various concentrations had been tabulated [27].

*(e) Lipid Peroxidation Inhibition Assay*. For this assay, egg yolk homogenate was used as lipid source and free radicals were produced by Fenton reagent (FeSO_4_/H_2_O_2_), a modified thiobarbituric acid reactive substances (TBARS) assay [28, 29]. In brief, 1 mL reaction mixture containing 0.5 mL egg yolk homogenate (10% in distilled water, v/v), 0.1 mL. of extract was mixed with 0.05 mL FeSO_4_ (0.07 M) and incubated for 30 min to induce lipid per oxidation. Free radical ruptures the lipid bilayer to form malonaldehyde as a secondary product. Two molecules of thiobarbituric acid react with one molecule of MDA to form pink colored product showing maximum absorbance at 532 nm called TBARS. When the reaction mixture was mixed with different concentrations of extracts of Meoh extract, it reduces the formation of TBARS product in concentration dependent manner in comparison to control (reaction mixture without antioxidant having absorbance 0.672 ± 0.033 at 532 nm).

*(f) Nitric Oxide Scavenging Ability*. Nitric oxide generated from aqueous sodium nitroprusside (SNP) solution interacts with oxygen to produce nitrite ions at physiological pH, which may be quantified and determined according to Griess Illosvoy reaction [30]. The reaction mixture contained: 10 mM SNP in 0.5 M phosphate buffer (pH 7.4) and various concentrations (100–1000 *μ*g/mL) of the MPE in a final volume of 3 mL. After incubation for 60 min at 37°C, Griess reagent (0.1% *α*-napthyl-ethylenediamine in water and 1% sulphanilic acid in 5% H_3_PO_4_) was added. The pink chromophore generated during diazotization of nitrite ions with sulfanilamide and subsequent coupling with *α*-napthyl-ethylenediamine were measured spectrophotometrically at 540 nm. Ascorbic acid was used as a positive control. Nitric oxide scavenging ability (%) was calculated by using above percent inhibition (*I*%) formula for DPPH assay.

*(g) Reducing Power*. Reducing power of MPE was determined according to the developed method [18]. A 2.5 mL solution of extract (100–800 mg/mL) was mixed with equal volume of phosphate buffer (0.2 M, pH 6.6) and 1% potassium ferricyanide and placed in water bath at 50°C for 20 min. Then it was cooled rapidly and 2.5 mL of 10% trichloroacetic acid was added and vortexed. This incubation mixture was centrifuged at 3,000 rpm for 10 min and its 5 mL supernatant was mixed with equal volume of distilled water and 1 mL of 0.1% ferric chloride. It was further incubated at room temperature for 10 min and absorbance was read at 700 nm. The reducing property of test sample was standardized against quercetin and expressed as difference in optical density (OD) from control as well as test as 0.1 and expressed as *μ*g/mL a high degree of absorbance indicate the stronger reducing power.

### 2.6. Determination of Hemolytic Activity of Extract on Human Red Blood Cells (hRBC)

Hemolytic assay of MPE was carried out and tested for hemolytic activities on human hRBC. Freshly collected human red blood cells were taken and washed three time by sterile phosphate buffer (PBS, NaCl (150 mM), NaH_2_PO_4_ (1.9 mM), and Na_2_HPO_4_ (8.1 mM), pH 7.4) at room temperature and resuspended in PBS four times its volume for subsequent analyses [31]. Each washing step was carried out by centrifuging the cells at 3000 rpm, 7 min, RT, discarding the supernatant after each wash. The serum was removed and the cells were resuspended to give a concentration of 5 × 10^8^ cells/mL PBS. Cell suspension was used throughout in the preparation of experimental and control tubes. Five different concentrations (20 *μ*g, 40 *μ*g, 60 *μ*g, 80 *μ*g, and 100 *μ*g) of extracts were mixed with 200 mL of RBC solutions and the final reaction mixture volume was made up to 1 mL by adding sodium phosphate buffer. The reaction mixture was then placed in water bath for 1 hour at 37°C. After the incubation time the reaction mixture was centrifuged again at 2500 rpm for 15 minutes. The supernatant was collected and the optical density was measured by reading well optical density at 541 nm using a multiwell plate Bio-Rad ELISA reader keeping sodium phosphate buffer as blank. Deionised water was used as a positive control. The experiment was done in triplicate and mean ± S.D. was calculated [32]:

(3) Percentage  hemolysis=Absorbance  of  sample−Absorbance  of  blankAbsobance  of  positive  control×100.

### 2.7. Statistical Analysis

The data was subjected to one-way analysis of variance (ANOVA) and the significance of the difference between means was calculated. Values expressed are mean of independent samples analyzed ± standard error of means (SEM). The data were subjected to correlation coefficient by using Sigmastat version 3.1 statistical analysis software. The correlation of the data was determined by Pearson's test. *P* values < 0.01, 0.05 were considered as statistically significant.

## 3. Results

For Phytochemical estimation, extract was filtered and solvent was removed by vacuum distillation. The extract was concentrated in a rotary evaporator and the residue was dried in desiccators over calcium chloride and weighed. The extract so obtained each time was mixed and later dried at 40°C in incubator. The final yield (w/w) of the extract was 11.6%. Enough quantity of the extract was prepared fresh before use.

### 3.1. Qualitative Phytochemical Estimation

MPE was examined for preliminary phytochemical screening through different standards showing the presence of alkaloids, phenolic groups, steroids, flavones, phenolic groups, saponins, steroids, sugars, tannins, and triterpenes in the phytochemical screening, on the basis of number of secondary metabolites (Table 1).

### 3.2. TLC and HPLC Analysis

Recent researches indicate that the polyphenols, being secondary metabolites, are present in rich amount in several plants. Many of them possess antioxidant, anti-inflammatory, and several other therapeutic properties. The result of TLC analysis using chloroform and methanol solvent mixture revealed five spots with following *R*_*f*_ values 0.81, 0.66, 0.63, 0.49, 0.31.

**Figure 1:**
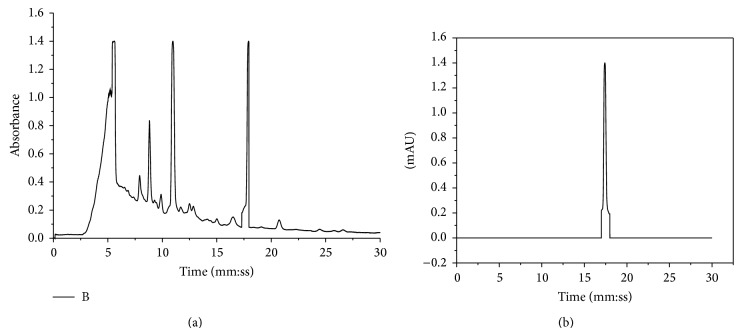
HLPC fringerprinting of (a) MPE compared to (b) standard rottlerin.

A correct assignment to the various compounds in the MPE was not possible. From UV spectra and retention times of the main peaks, some compound classes contained in the extract have been determined. High-performance liquid chromatography (HPLC) revealed the presence of polyphenol, rottlerin compared to standard used in the analysis (Figure 1). The precision as well as the reproducibility of this method were satisfactory. Quantitative HPLC studies show that the Kamala contained Rottlerin (21.19%, w/w) very rich quantity, with retention time in minute (*R*_*t*_ value 17.74) recorded comparable to standard used. Although a primary objective of carrying out HPLC was to standardize the extract used in different pharmacological activities.

### 3.3. Quantitative Estimation of MPE

Total phenolic content was expressed in *µ*g of gallic acid equivalent. Phenolic compounds may contribute directly to antioxidative action. The total phenolic content was 21.25 ± 1.5 mg/g of gallic acid equivalent per mg extract in hydro alcohol. All values are expressed in mean ± SEM (*n* = 4).

The total flavonoid content of MPE was 43.26 ± 2.66 mg/g quercetin equivalent per mg plant extract. All values are expressed in mean ± SEM (*n* = 4).

### 3.4. DPPH Radical Scavenging Activity

**Figure 2:**
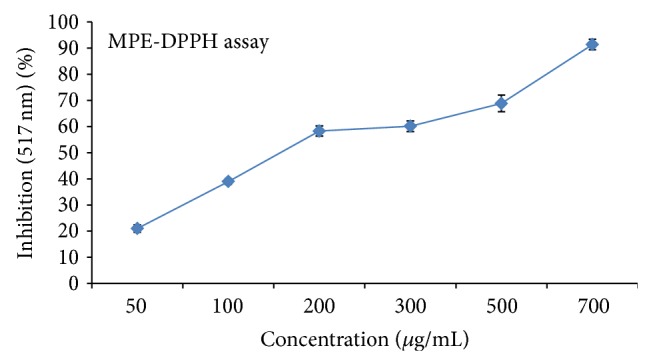
Trapping potential for DPPH radicals scavenging activity of fruit extract of* M. philippenensis*. Levels of significance: ^*^ *P* < 0.05 and ^**^ *P* < 01.0. The absorbance of only DPPH solution at 517 nm was 0.645 ± 0.032 (experimental control). Trapping potential of BHT-EC_50_ 28.61 ± 1.40.

*In vitro* antioxidant assay of MPE revealed the presence of antioxidant potential. The percentage of inhibition was observed in all the antioxidant models that free radicals were scavenged by the plant extract in a concentration dependent manner up to the given concentration. The percentage inhibition of scavenging activities of the MPE for DPPH showed 58.30% DPPH inhibition at 200 *µ*g/mL concentrations. Antioxidant activity depends on the presence of amount of total polyphenolic compounds (Figure 2).

### 3.5. ABTS Radical Scavenging Activity

**Figure 3:**
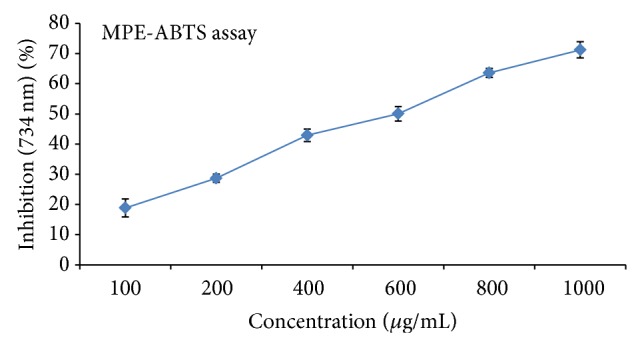
Graphical presentation of different concentrations of MPE on ABTS^∙+^ radicals in terms of percent inhibition. Levels of significance: ^*^ *P* < 0.05 and ^**^ *P* < 0.01. The absorbance of only ABTS solution at 734 nm was 0.712 ± 0.032 (experimental control). Trapping potential of ascorbic acid-EC_50_ 33.81 ± 2.40.

The total antioxidant activity of the extract was calculated from the decolorization of ABTS^∙+^, which was measured spectrophotometrically at 734 nm. Interaction with the extract or standard Vitamin C (50 *µ*g/mL) suppressed the absorbance of the ABTS^+^ radical cation and the results are expressed as percentage inhibition of absorbance. The result showed that different concentrations of MPE showed varying degree of scavenging potential for ABTS^∙+^ radicals in concentration dependent manner. Thus, based on their EC_50_ value total methanolic fraction of was found to be most active, when MPE was compared with the trapping potential of ascorbic acid. Maximum inhibition of 71.21 ± 2.67 at 1000 *µ*g/mL among different concentrations of 100, 200, 400, 600, 800, and 1000 *µ*g/mL with EC_50_ 275.29 ± 3.85 (Figure 3).

### 3.6. Superoxide Radical Scavenging Activity

**Figure 4:**
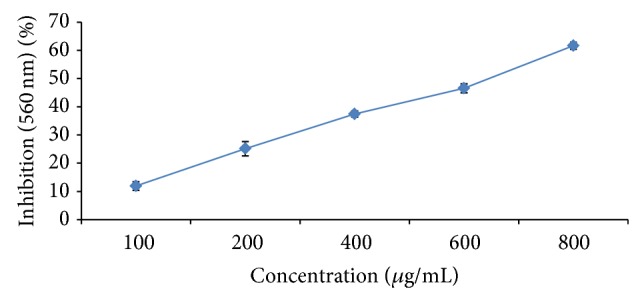
Superoxide radical scavenging activity of MPE. Levels of significance: ^*^ *P* < 0.05 and ^**^ *P* < 0.01. Trapping potential of copper sulphate-EC_50_ 44.90 ± 1.40.

The ability to reduce NBT by PMS-NADH coupling can measure the superoxide radicals generated from dissolved oxygen. The decrease in absorbance at 560 nm with the MPE and the reference compound copper sulphateindicates their abilities to quench superoxide radicals in the reaction mixture. Superoxide free radicals showed maximum inhibition of 72.45 ± 3.19 at concentration of 1000 *µ*g/mL plant extract with EC_50_ value of 325.24 ± 5.79 proving again the better antioxidant activity. As shown in Figure 4, the EC_50_ values of the copper sulphate on superoxide scavenging activity was 44.90 ± 1.40 *μ*g/mL (Figure 4).

### 3.7. Hydroxyl Radical Scavenging Activity

Result shows the abilities of the extract and standard thiourea to inhibit hydroxyl radical-mediated deoxyribose degradation in a FeCl_3_-EDTA-ascorbic acid and H_2_O_2_ reaction mixture. The EC_50_ values of the MPE for scavenging hydroxyl radicals are 415.21 ± 2.08 *μ*g/mL in presence of EDTA with maximum inhibition of 55.56 ± 1.22 *μ*g/mL at concentration of 1000 *μ*g/mL and EC_50_ value of 122.19 ± 1.41 *μ*g/mL in absence of EDTA with maximum inhibition of 93.89 ± 3.98 *μ*g/mL at concentration of 1000 *μ*g/mL. EC_50_ value of standard, thiourea used in assay is 315.5 ± 0.08 (Table 2).

### 3.8. Lipid Peroxidation Inhibition Assay

**Figure 5:**
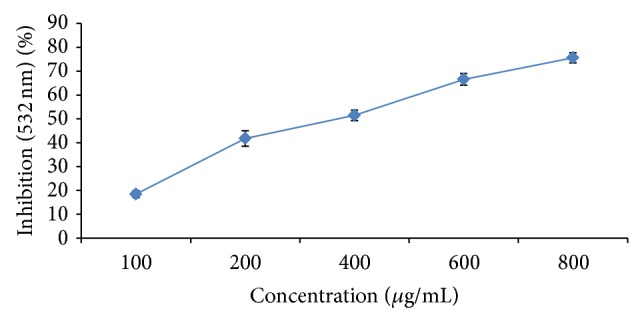
Effect of MPE on FeSO_4_ induced lipid per oxidation in egg yolk homogenate. Levels of significance: ^*^ *P* < 0.05 and ^**^ *P* < 0.01. Result was expressed in terms of % reduction in lipid per oxidation in comparison to control (reaction mixture without antioxidant having absorbance 0.672 ± 0.033 at 532 nm). EC_50_ 27.12 ± 0.12.

Result of antilipoperoxidation free radicals of MPE to prevent peroxidation showed that maximum trapping potential for LPO radicals is 87.36 ± 2.19 at the concentration of 1000 *µ*g/mL with EC_50_ 635.53 ± 6.12 (Figure 5). Oxidative stress in cells and tissues can be best monitored by its lipid per oxidation assay, a well-established mechanism both in plants and animals.

### 3.9. Nitric Oxide Scavenging Ability

**Figure 6:**
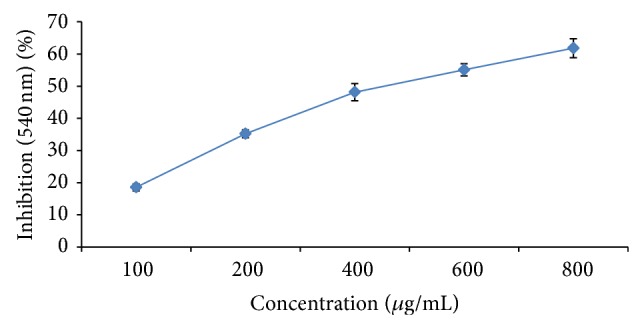
Effect of MPE on trapping potential for nitric oxide radicals. Level of significance: ^*^ *P* < 0.05, value is highly significant ^**^ *P* < 0.01 in comparison to control. Ascorbic acid was used as a reference compound and its EC_50_ value is 11.6 *μ*g/mL.

MPE caused a moderate dose-dependent inhibition of nitric oxide with an EC_50_ of 275.19 ± 3.5 *μ*g/mL (Figure 6). Ascorbic acid was used as a reference compound and its IC_50_ value is 11.6 *μ*g/mL. At 800 and 1000 *μ*g/mL, the percentage inhibition of the plant extract was significant, that is, 55.06 ± 1.86 and 61.78. ± 1.98, respectively, whereas that of ascorbic acid was 11.60%.

### 3.10. Reducing Power

**Figure 7:**
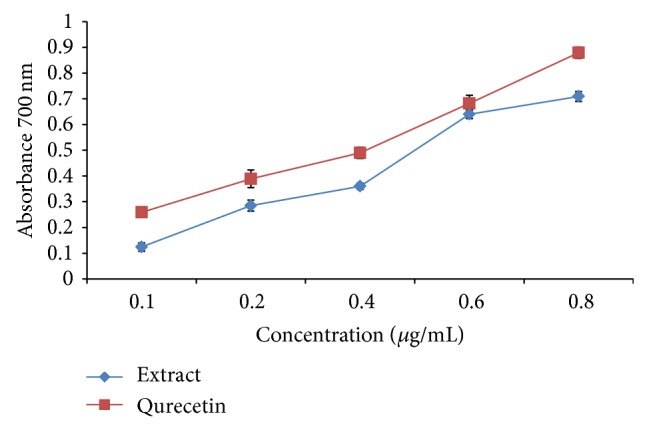
Reducing power of MPE in hydroalcoholic extract and comparison with quercetin. Reducing power assay shows the abilities of* Mallotus* extract and the standard quercetin. The absorbance (A700) was plotted against concentration of sample. Each value represents mean ± S.D. (*n* = 6). ^***^ *P* < 0.001 versus 0.6 mg/mL.

As illustrated in Figure 7, Fe^3+^ was transformed to Fe^2+^ in the presence of MPE and the reference compound quercetin to measure the reductive capability. At 0.1 mg/mL, the absorbances of the plant extract and quercetin were 0.124 ± 0.016 and 0.259 ± 0.016, respectively, while at 0.6 mg/mL, the absorbances of both extract and BHT were almost the same. This result indicates that maximum activity is shown at this dose by the extract.

### 3.11. Antihemolytic Activity

**Figure 8:**
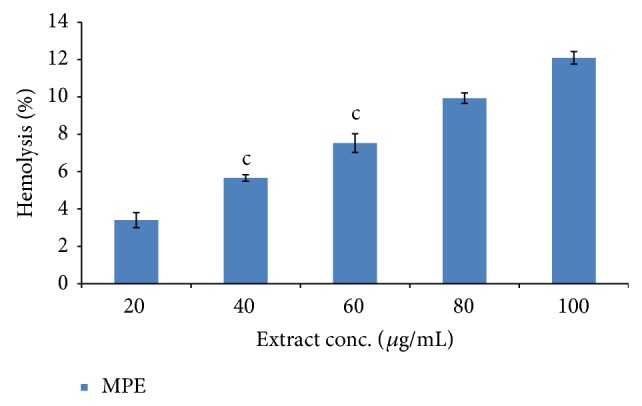
Antihemolytic activity of fruit extract of* M. philippenensis*. Each value is represented as mean ± SE (*n* = 3). Values at concentrations 40 and 60 are significantly indicated by letter c (*P* < 0.05) compared to positive control values.

Hemolytic activity of MPE was screened against normal human erythrocytes. MPE with different concentrations exhibited differential pattern hemolytic effect towards human erythrocytes. Result indicated that the extract of plant exhibits minimum hemolytic activity, showing antihemolytic behavior. Lysis of erythrocytes was found to be increased with an increase of extract concentration (Figure 8).

## 4. Discussion

Herbal medicine and its derived products has been the mainstay of traditional medicines around the world. The phytochemicals present in the plant and their food products are generally nontoxic and have the capacity to prevent chronic diseases. Generally, the plant products encompass high concentration of flavonoids and phenolic content. Flavonoids play a vital role in protection against human diseases like lipid peroxidation involved in atherogenesis, thrombosis, carcinogenesis, hepatotoxicity, and a variety of disease conditions [33]. The HPLC analysis of MPE shows the presence of rottlerin and other important peaks of phenols and flavonoids, which are reported to have anti-inflammatory, antiasthmatic, analgesic, and antioxidant activity and these findings are in concordance with our results. Most of the medicinal plants contain flavonoids, which have been shown to have diuretic, laxative, antispasmodic, antihypertensive, and anti-inflammatory actions. Flavonoids and saponins are well known for their anti-inflammatory ability due to their inhibitory effects on enzymes involved in the production of the chemical mediator of inflammation.

Free radicals are generated continuously in the human body due to metabolism and diseases [34]. They can cause extensive damage to tissues and biomolecules leading to various disease conditions, especially degenerative diseases and extensive lysis [35]. Oxidative damage can be overcome by many synthetic drugs available but they are associated with adverse side effects. Alternate solution to the above side effects is to consume natural antioxidants from food supplements and traditional medicine [36–38]. Till date, many natural antioxidants has been isolated and reported for their activity [39]. In order to protect from free radicals, organism has endowed with endogenous (catalase, superoxide dismutase, glutathione peroxidase/reductase) and exogenous (Vitamin C and E, carotene, uric acid) defense systems, but these systems will not work in some critical conditions such as oxidative stress, contamination, UV exposure, microbial infections, where the production of free radicals significantly increases [40]. There is increasing demand of indigenous medicine in the protective biochemical functions of natural antioxidants contained in spices, herbs, and medicinal plants.

Due to the complex nature of phytochemicals, a single method to evaluate the antioxidants activity cannot be evaluated. In this context, different standard methods were used to validate nature of plant extract in terms of antioxidants. The phytochemical analysis conducted on* M. philippenensis* fruit extract revealed the presence of tannins, flavonoids, steroids, and saponins. The results indicate that* Mallotus* fruit extract contains significant amounts of flavonoids and phenolic compounds. Both these classes of compounds have good antioxidant potential and their effects on human nutrition and health are considerable. The mechanism of action of flavonoids is through scavenging or chelation [41] and its effects on membrane permeability also act on membrane-bound enzymes like ATPase and phospholipase [42] which explains the antioxidant mechanism of* Mallotus* fruit extract. It supports its use in stress related diseases or uses in wound dressing, cuts, and sores [43]. Flavonoids due to actions by its anion radicals serve as health promoting compound [44]. Phenolic compounds are also very important plant constituents because their hydroxyl groups confer scavenging ability.

The plant extract was able to reduce the stable free radical of DPPH to the yellow coloured diphenylpicrylhydrazine. This proofs that the* Mallotus* fruit extract contains some active constituents that are capable of donating hydrogen to a free radical in order to remove odd electron which is responsible for radical's reactivity. DPPH radical scavenging method has been proven to be good because its results are not affected by substrate polarity. Scavenging ability of the* Mallotus* fruit extract shows the potential decrease in the concentration of DPPH.

The scavenging activity of ABTS^+^ radical by the plant extract was found to be appreciably reported, as ABTS^+^ is a blue chromophore produced by the reaction between ABTS and potassium persulfate. Plant extract while addition to this preformed radical cation reduced it to ABTS in a concentration-dependent manner. The results were compared with those obtained using standard Vitamin C and the TEAC value demonstrates that the extract is having potent antioxidant [45].

Superoxide anion radical is one of the strongest reactive oxygen species among the free radicals [46] and also very harmful to cellular components [47]. Robak and Gryglewski [48] reported that flavonoids are found to be most effective antioxidants mainly because they can easily scavenge superoxide anions. The results suggest that radical scavenging effect is increased of both extract and reference compound with increases in concentration, showing that plant extract is a more potent scavenger of superoxide radical than the standard copper sulphate.

In living organisms, hydroxyl radical and superoxide radical are being continuously formed in a process of reduction of oxygen to water. Hydroxyl radicals are the major active oxygen species causing lipid peroxidation and enormous biological damage like reduction of disulfide bonds in proteins, specifically fibrinogen, resulting in their unfolding and scrambled refolding into abnormal spatial configurations [49]. Consequences of this reaction are observed in many diseases such as atherosclerosis, cancer, and neurological disorders, and can be prevented by the action of nonreducing substances [50]. When* Mallotus* fruit extract was added to the reaction mixture, it removed the hydroxyl radicals from the sugar and prevented the reaction. The IC_50_ value indicates that the plant extract is a better hydroxyl radical scavenger than the standard mannitol.

Lipid per oxidation inhibition assay of plant extract inhibits the FeSO_4_ induced lipid peroxidation in egg yolk, which is the net result of iron-mediated hydroxyl radicals. This can be achieved either by scavenging the hydroxyl radicals or by chelating the iron ions, which is responsible for initiation of Fenton's reaction. Earlier studies with the plant of* Mallotus philippenensis* indicate good antioxidant and radical scavenging properties [10]. Different researchers reported the presence of tannins which have metal chelating and hydroxyl radical scavenging properties [51].

Nitric oxide (NO) is a reactive free radical produced by phagocytes and endothelial cells and plays an important role in inflammatory process. The sustained level of nitric oxide is directly toxic to tissue and causes injury to cell leading to vascular collapse whereas its high level which causes chronic expression of nitric oxide radical is associated with various carcinomas and inflammatory conditions including juvenile diabetes, multiple sclerosis, arthritis, and ulcerative colitis [52]. The toxicity of NO increases greatly when it reacts with superoxide radical, forming the highly reactive peroxynitrite anion (ONOO^−^) [53]. In this study, level of nitric oxide was significantly reduced by* Mallotus* extract, explaining its role in treatment of inflammation and for wound healing [54].

Reducing power of the extract was compared with standard quercetin and found to be superior indicating its potential antioxidant behavior. As shown in Figure 7,* Mallotus* exhibits significant reducing power as it possesses various mechanisms such as prevention of chain initiation, decomposition of peroxides, reducing capacity, and radical scavenging.

The protective effect of* Mallotus* fruit extract has been evaluated on oxidative damage of erythrocytes membrane (lipid and protein peroxidation) which may be implicated in hemolysis [55]. Erythrocytes were considered to be the major target of free radicals generated while redox reaction and high content of polyunsaturated fatty acids [56]. Deionized water was used as a positive control due to its oxidative nature with respect to destruction of cell membrane. In the present study,* Mallotus* extract exhibited potent antihemolytic activity at different concentrations and activity decreases while increasing the extract concentration which could be due to the presence of above reported phytochemicals in concentrated form of fruit extract. It concludes that extract contains some molecules which interacted with a class of lipids present in the outer monolayer of the human erythrocyte membrane showing protective effect. As far as safety profile of extract is concerned, alcoholic extract of* Mallotus philippenensis* fruit extract (MPE) was found safe at the dose of 2000 mg/kg according to OECD guidelines 425 [57, 58].

## 5. Conclusion

On the basis of the above experiments of antioxidant assay in different models, it is concluded that extract of* Mallotus philippenensis* fruit contains large amounts of phenolic and flavonoids compounds and exhibits high antioxidant and free radical scavenging activities. It also exhibits reducing power. These* in vitro* assays of antioxidant indicate that this plant extract is a significant source of natural antioxidant, which might be helpful in preventing the progress of various oxidative stresses. We are reporting first time the protective effect of fruit extract on haemolysis activity which may be attributed due to the presence of tannins, phenols, and good nature of antioxidants but the mechanism is still unclear. However, the components responsible for the above mentioned activity are currently unclear. Therefore, further investigation is needed to isolate, purify and identify the pure moiety responsible for antioxidant and protective effect in the plant. Furthermore, the* in vivo* antioxidant activity of this extract needs to be assessed prior to clinical use.

## Figures and Tables

**Table 1:** Phytochemical tests of *Mallotus philippenensis* fruit extract.

S. No.	Constituents	Tests	Hydroalcoholic extract (MPE)
1	Alkaloids	Mayer's reagent	++
		Dragendroff's reagent	+++
		Hager's reagent	++
		Wagner's	−

2	Sterols	Liebermann's sterol test	+
		Liebermann's test	+
		Salkowski's test	+

3	Carbohydrate and glycosides	Molisch's reagent	+++
		Fehling's reagent	++
		Barfoed's reagent	++
		Borntrager's reagent	+
		5% KOH	−

4	Fixed oils and fats	Spot test	−
		Saponification	−

5	Phenolic compounds	Extract + Fecl_3_	+++

6	Test for tannins	Lead acetate solution	+
		Fecl_3_	−

7	Proteins and amino acids	Biuret test	++
		Ninhydrin test	++
		Xanthoproteic test	++
		Million's reagent	++

8	Triterpenoids and saponins	Tin + Thionyl Chloride	++
		Foam test	+
		Haemolysis test	+

9	Gums and mucilages	Precipitation with 95% alcohol	−
		Molisch's test	−
		Ruthenium test	−

10	Flavone and flavonoids	Aqueous NaOH	+++

+: present, ++: present at moderate level, +++: present at high level, and −: absent.

**Table 2:** Effect of MPE for scavenging hydroxyl radicals non-site-specific hydroxyl radical-mediated 2-deoxy-dribose degradation (in presence/absence of EDTA) in terms of % inhibition (absorbance at 532 nm).

Concentration MPE (*μ*g/mL)	% of inhibition in absorbance at 532 nm (mean ± SD) *n* = 6	
	In presence of EDTA	In absence of EDTA
100	13.83 ± 1.02	40.11 ± 1.08
200	27.72 ± 1.45	57.60 ± 1.21
400	34.54 ± 1.65	70.88 ± 1.68
600	40.14 ± 2.34	75.23 ± 1.86
800	51.25 ± 2.67^*^	80.78. ± 1.98^*^
1000	55.56 ± 1.22^*^	93.89 ± 3.98^*^
EC_50_	415.21 ± 2.08	122.19 ± 1.41

Values are mean ± SEM of 6 experiments in each group.

Level of significance: ^*^ *P* < 0.05. Result was expressed in terms of % inhibition in comparison to % control values. Trapping potential of thiourea in presence and absence of EDTA-EC_50_ 42.76 ± 2.09 and 77.28 ± 1.16, respectively.
